# Non‐Syndromic Paucity of Interlobular Bile Ducts (NSPIBD) Presenting as Neonatal Cholestasis in an Infant With Down Syndrome: A Case Report

**DOI:** 10.1002/ccr3.72060

**Published:** 2026-03-16

**Authors:** Pui Ling Thong, Nurfirdaus Musa, Nurul Husna Mohd Dani, Zurina Zainuddin

**Affiliations:** ^1^ Department of Paediatrics, Paediatric Gastroenterology, Hepatology and Nutrition Unit Hospital Sultan Abdul Aziz Shah, Universiti Putra Malaysia Selangor Malaysia; ^2^ Department of Paediatrics Hospital Sultan Abdul Aziz Shah, Universiti Putra Malaysia Selangor Malaysia; ^3^ Department of Pathology Faculty of Medicine, Universiti Kebangsaan Malaysia Kuala Lumpur Malaysia

**Keywords:** down syndrome, neonatal cholestasis, non‐syndromic paucity of interlobular bile ducts, pale stool

## Abstract

Non‐syndromic paucity of interlobular bile ducts (NSPIBD) is a rare cause of neonatal cholestasis, often presenting with clinical features similar to extrahepatic biliary atresia. This report presents a case of NSPIBD in an infant with Down syndrome who exhibited jaundice and pale stools. Ultrasonographic findings and an inconclusive cholangiogram raised concern for BA, yet liver histology demonstrated preserved bile flow and bile duct paucity without ductular proliferation or portal fibrosis. This highlights the necessity of a multimodal diagnostic approach, integrating clinical course, biochemistry, imaging, and histology, particularly when findings are discordant. Management was primarily supportive, focusing on cholestasis treatment, and the prognosis remains variable. Therefore, NSPIBD should be recognized as a significant differential diagnosis of cholestasis in infants with Down syndrome, and early diagnosis is essential to avoid unnecessary surgical intervention.

## Key Clinical Message

Clinicians should consider non‐syndromic paucity of interlobular bile ducts as a differential diagnosis for cholestasis in infants with Down syndrome. Its presentation often mimics biliary atresia, making early liver biopsy essential to avoid unnecessary surgery.

## 1 Introduction

The incidence and prevalence of non‐syndromic paucity of interlobular bile ducts (NSPIBD) remain undetermined [1]. Clinically, NSPIBD closely resembles extra‐hepatic biliary atresia, as both conditions manifest with neonatal cholestasis and pale stools. Histopathological examination typically demonstrates portal changes, including bile duct paucity and fibrosis, as well as lobular alterations such as cholestasis, giant cell transformation, extramedullary hematopoiesis, and perisinusoidal fibrosis [2]. This case report describes the clinical presentation spectrum of NSPIBD in an infant with Down syndrome and examines the disease course and outcomes in this patient population. Management primarily involves supportive care for cholestasis, and prognosis varies among affected infants.

## 2 Case History and Examination

A term male infant with Down syndrome was admitted to the neonatal intensive care unit after delivery due to presumed sepsis and feeding intolerance. He was started on empirical antibiotics with intravenous penicillin and gentamicin. The septic workup, which included blood culture, yielded negative results. At 2 weeks of age, he developed prolonged jaundice, which was associated with intermittent dark urine and acholic stools. He had no prior history of receiving parenteral nutrition. Laboratory results showed elevated serum bilirubin, with a conjugated fraction of 45% (total bilirubin 213 μmol/L, direct 96 μmol/L). The infant also exhibited transient abnormal myelopoiesis, evidenced by the presence of non‐erythroid blasts constituting 8% of the peripheral blood film. An echocardiography revealed a small patent foramen ovale.

Upon clinical examination, the infant appeared jaundiced and pale, with no lymphadenopathy or peripheral signs of chronic liver disease. The abdomen was not distended, but the liver and spleen were palpable at 5 and 3 cm, respectively, below the subcostal margin. An audible murmur was detected at the left sternal edge with a non‐displaced apex beat. Other systemic examinations were unremarkable.

Laboratory tests indicated a hemoglobin level of 14.4 g/dL, white blood cell count 5000/mm^3^, platelet count 91,000/mm^3^, and 8% blast cells on the peripheral smear. Coomb's test, C‐reactive protein, and thyroid function tests were normal. At 4 weeks of age, total bilirubin was 177 μmol/L, with a conjugated fraction of 48%. Serum levels of AST, ALT, and GGT were 128 U/L, 106 U/L, and 253 U/L, respectively. Given the persistent clinical signs of pale stools and abnormal biochemistry indicative of cholestasis, further evaluation was warranted to identify the underlying cause. Table 1 outlines the trends in liver biochemistries and other laboratory investigations to exclude various causes of cholestasis.

**TABLE 1:** Summary of laboratory investigations.

Investigations	Week 2	Week 4	Week 5	Week 6	Week 20	Normal values
Bilirubin, μmol/L (conjugated fraction, %)	213 (45)	177 (48)	190 (55)	171 (57)	28	< 17
Aspartate aminotransferase, U/L		128	190	400	141	20–60
Alanine aminotransferase, U/L		106	265	289	228	10–39
Alkaline phosphatase, U/L		686	681	396	701	150–507
Gamma‐glutamyl transpeptidase, U/L		203		253	636	12–23
Albumin, g/L		42	37	40	34	28–47
International normalized ratio		1.1			0.93	0.8–1.2
Partial thromboplastin time, s		12.0			10.2	25–36.3
Alpha‐1‐antitrypsin, g/L		1.1				0.9–2.0
Anti‐rubella IgG, IU/mL Anti‐CMV IgG Anti‐CMV IgM Anti‐toxoplasma IgG Anti‐toxoplasma IgM	Non‐reactive					
Urine succinylacetone Galactosemia screen Urine organic acids Serum amino acids		Non diagnostic profile				
Urine culture		No growth				
Karyotype 47, XY, +21						
Vitamin D, nmol/L					11	< 50

**FIGURE 1:**
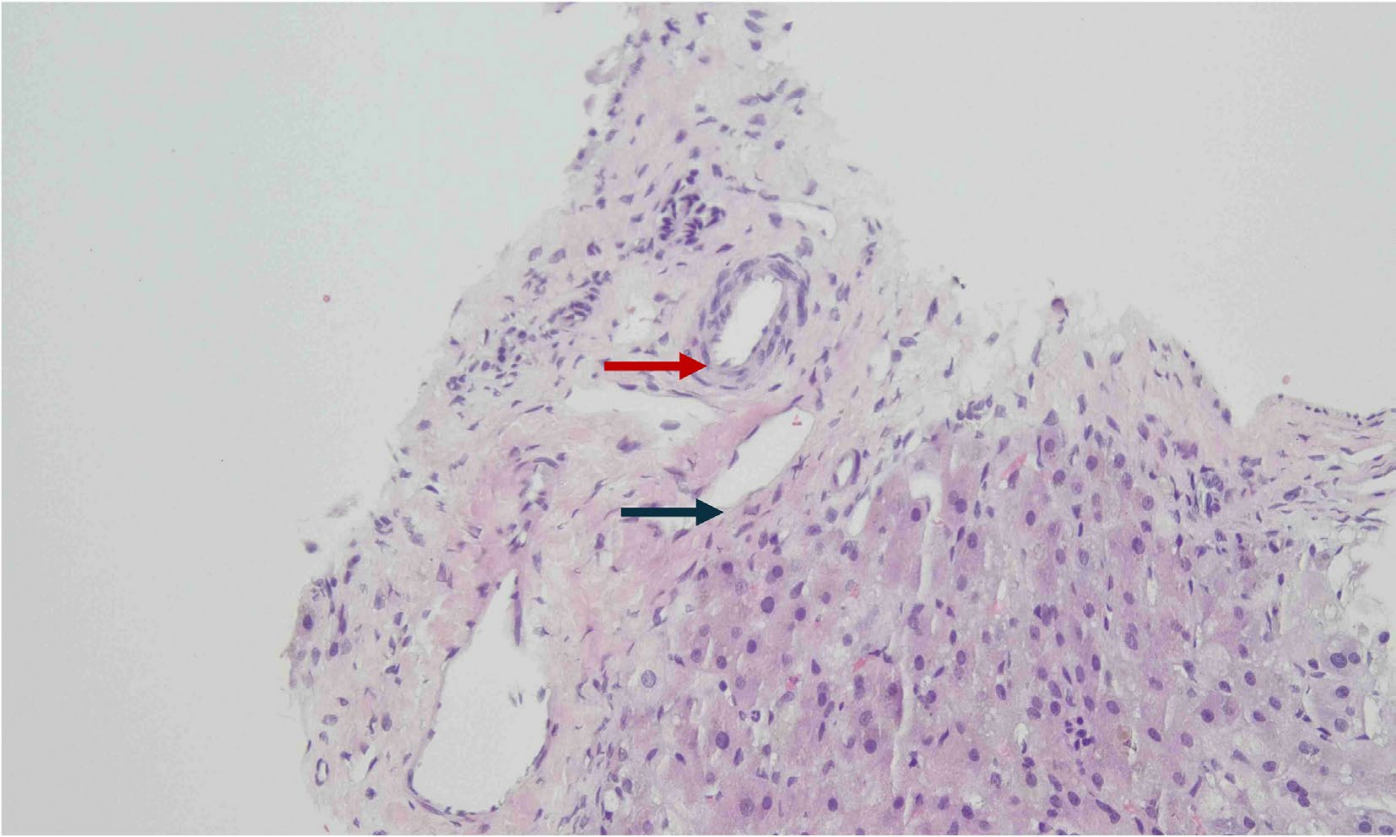
Portal vein (blue arrow) and portal artery (red arrow), with absence of bile duct (Hematoxylin & Eosin ×400).

**FIGURE 2:**
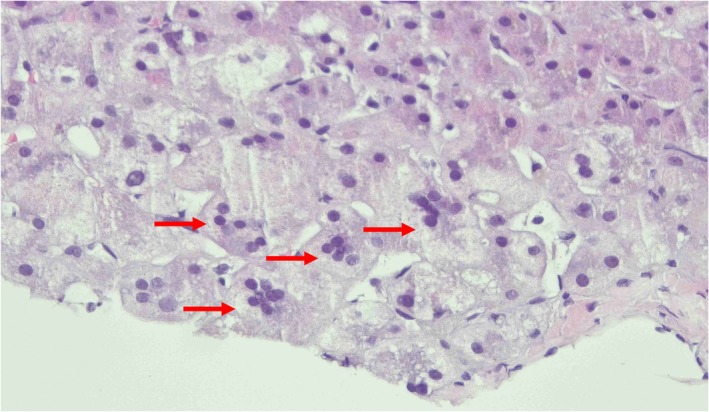
Hepatocytes with giant cell transformation (red arrow) (Hematoxylin & Eosin ×400).

**FIGURE 3:**
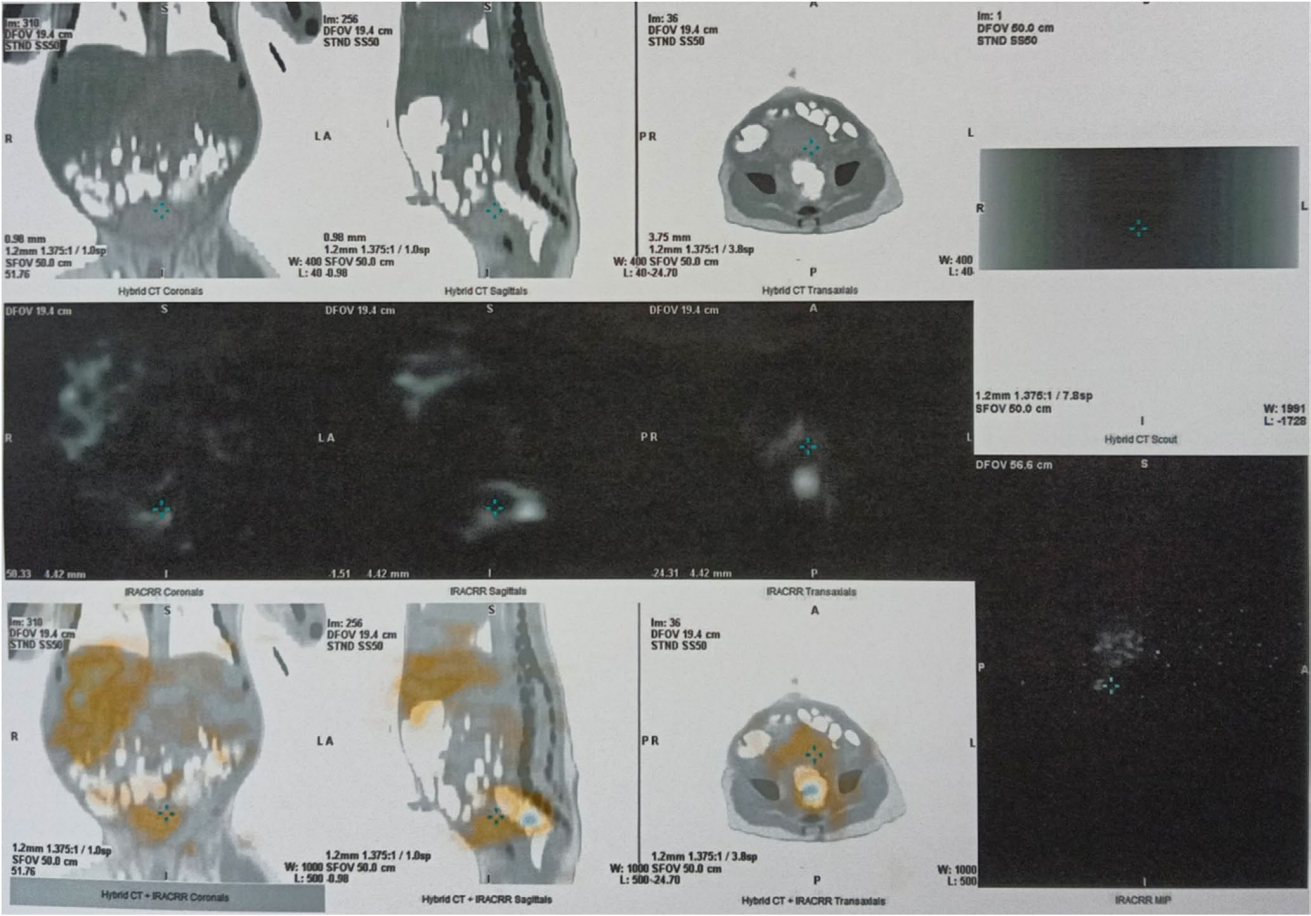
Good and homogenous tracer uptake was seen in the liver with satisfactory pool clearance. Faint tracer accumulation is seen in the lower abdominal region at 24 h of the study, with intraluminal uptake observed in the rectosigmoid colon on SPECT–CT.

Abdominal ultrasonography revealed a type II gallbladder characterized by a thickened wall and hyperechogenic mucosal lining. Additionally, there was an abnormal hepatic artery to portal vein ratio of 0.47. Given the potential diagnosis of biliary atresia, a percutaneous transhepatic cholangiogram was performed when the patient was 5 weeks old. This procedure showed the contrast confined to a well‐distended gallbladder; however, it indicated a lack of flow into both the common bile duct and common hepatic duct due to a contrast leak, resulting in inconclusive findings. A liver biopsy was performed the following day, obtaining a good core sample that included nine portal tracts. The hepatic histology revealed features consistent with bile duct paucity (Figure 1) and the presence of giant cell hepatocytes (Figure 2). There was no significant ductular proliferation or portal fibrosis. These findings are indicative of NSPIBDs. Additionally, a hepatic scintigraphy was also performed, which showed good homogeneous tracer uptake in the liver, followed by satisfactory pool clearance. Intraluminal tracer uptake was visualized in the rectosigmoid area 24 h after the study, which helped to exclude the possibility of biliary atresia (Figure 3). The treatment plan included initiating ursodeoxycholic acid therapy and providing supplementation of fat‐soluble vitamins. The patient was discharged home at 6 weeks of age.

## 3 Outcome and Follow‐Up

Over the next few months, his jaundice, bilirubin levels, and liver enzymes gradually improved. His stool color normalized around 3 months of age. Upon review in clinic at 5 months old, his biochemical indices demonstrated an improvement in the serum bilirubin and aminotransferase levels. He remained well during his recent clinic visit, and his interim growth was satisfactory.

## 4 Discussion

Infants with Down syndrome have an increased risk of developing cholestasis, which can result from various causes. A retrospective study involving 206 infants with Down syndrome found that 3.9% developed neonatal cholestasis [3]. The term NSPIBD in infants with Down syndrome refers to a condition characterized by a reduced number of interlobular bile ducts in liver histology, occurring without the typical features of Alagille syndrome. Although this condition is not commonly seen in infants with Down syndrome, it is a recognized association. Puri et al. [4] first described paucity of interlobular bile ducts in infants with Down syndrome in 1975, followed by Kahn et al. [2], who reported a series of 17 cases of NSPIBD, including two cases involving Down syndrome, in 1986.

The precise mechanism by which NSPIBD causes neonatal cholestasis in infants with Down syndrome is not fully understood. None of the known controlling genes associated with the uptake, synthesis, or secretion of bile acids in cholangiocytes or enterocytes has been mapped to chromosome 21. While specific genes on chromosome 21 responsible for NSPIBD in infants with Down syndrome remain unidentified, it is hypothesized that overexpression of specific genes may disrupt the cell signaling pathway, adhesion, and development of the bile ducts, potentially impairing their formation and maintenance [5]. Furthermore, the presence of an extra chromosome 21 has been linked to altered inflammatory responses in the liver, which may contribute to bile duct destruction [6].

NSPIBD in infants with Down syndrome typically presents conjugated hyperbilirubinemia within the first month of life. It is associated with other symptoms, including acholic stools, pruritus, poor growth, and malabsorption. Clinicians evaluating cholestatic jaundice in this patient population should consider a broad differential diagnosis, including congenital infections (such as cytomegalovirus and rubella), endocrine abnormalities (like hypopituitarism and hypothyroidism), transient myeloproliferative disorder, and anatomical obstructions. Although cases of biliary atresia have been reported in individuals with Down syndrome, it is not a common cause of cholestasis in these infants. Kotb et al. [7] found that none of the infants with Down syndrome who experienced cholestasis had biliary atresia. Some studies suggest that the compromised neutrophil function associated with Down syndrome may offer a protective effect against the development of biliary atresia.

In our present case, cholangiography and liver biopsy were performed at 5 weeks of age (following inconclusive findings of both ultrasonography and cholangiography), which also endeavors to exclude other possible causes of cholestasis, particularly transient myeloproliferative disorder. The liver biopsy revealed hallmark findings of bile duct paucity, variable portal fibrosis, lobular changes with giant cell hepatocytes, and extramedullary hematopoiesis—findings similar to those observed in other case reports (Table 2). Bile duct paucity is defined as a ratio of interlobular bile ducts to portal tracts that is less than 0.5 in at least five well‐defined portal tracts [8]. The number of interlobular bile ducts might appear normal in early biopsy specimens due to the variable time course of the destructive process. In some patients, the ducts disappear by 4–5 weeks of age, while in others, duct paucity may only become evident after 3 months. Early portal changes in liver biopsies can help distinguish between syndromic and non‐syndromic forms of bile duct paucity in infants. In this patient, the presence of early portal changes appears more characteristic of the non‐syndromic form, suggesting a primary insult to the bile ducts [4]. A liver biopsy is also crucial for confirming the diagnosis of biliary atresia. The most informative histological features include prominent bile ductular proliferation and portal fibrosis; however, in this case, neither feature was present. While these changes are not specific, they can help to exclude the diagnosis of biliary atresia when interpreted in the appropriate clinical context and with supportive radiological tools.

**TABLE 2:** Cases of NSPIBD in Down syndrome reported in the past and their characteristics.

Author/year	Age	Sex	Clinical presentation	Liver biopsy timing	Histology results	Treatment	Outcomes
Chang et al., 1999	1 week old	Female	Jaundice, acholic stools, hepatomegaly	3 weeks of life	Bile duct paucity, cholestasis within hepatocytes and biliary canaliculi, bile duct proliferation, giant cells hepatocytes and fibrosis	Phenobarbital, ursodeoxycholic acid	Resolved jaundice and acholic stools at 5 months old
MacPherson et al., 2015	1 month old	Male	Jaundice, dark urine, acholic stools, hepatomegaly	4 weeks of life	Intrahepatic cholestasis, bile duct paucity in the portal areas, small bile ductule with absence of bile plugs, early bile ductular reaction in the periportal areas	Ursodeoxycholic acid and fat‐soluble vitamin supplements	Resolved jaundice and acholic stools at 3 months old
Kahn et al., 1986	2 patients, 4–6 weeks old	Unknown	Jaundice, acholic stools	< 90 days	Bile duct paucity, giant cell transformation, extramedullary hematopoeisis	Ursodeoxycholic acid	Resolved jaundice and acholic stools
Kotb et al., 2019	18 patients, neonates and infants	Unknown	Jaundice, acholic stools	< 90 days	Paucity of bile ducts, diffuse hepatocyte ballooning, infiltration with inflammatory cells, bile duct proliferation.	Ursodeoxycholic acid	17 patients cleared cholestasis. 1 patient progressed to chronic liver disease

Alagille et al. reported that NSPIBD generally has a more severe course compared to syndromic forms [9]. While some reports indicate high mortality rates associated with the non‐syndromic form, specific percentages regarding progression to cirrhosis versus benign resolution in the context of Down syndrome are not consistently documented across large‐scale studies. Currently, the prognosis for NSPIBD remains unclear, with reported outcomes varying from progressive cirrhosis to benign resolution (Table 2). Much of the existing literature is based on case reports, which underscores the necessity for more comprehensive cohort studies to determine the precise outcomes and prognosis for this condition in infants with Down syndrome. The progression of bile duct paucity in this population is complex and may be affected by various factors, including the presence of other comorbidities. NSPIBD should be suspected in infants with Down syndrome who exhibit consistent clinical features, and further evaluation should be pursued in an appropriate clinical context.

## 5 Conclusion

In summary, NSPIBD should be considered in infants with Down syndrome who have persistent cholestasis and pale stools, particularly when imaging does not show evidence of extra‐hepatic biliary atresia. When imaging results are inconclusive, a liver biopsy is recommended, as interpreting these investigative tools can be challenging with consideration of the possible potential pitfalls in interpretation. Prompt and accurate diagnosis is essential to avoid unnecessary surgical interventions. Since NSPIBD is a rare condition with a variable outcome, more case studies are needed to better understand its association with Down syndrome as a potential cause of neonatal cholestasis and to monitor its prognosis.

## Author Contributions

**Pui Ling Thong:** conceptualization, data curation, formal analysis, investigation, writing – original draft, writing – review and editing. **Nurfirdaus Musa:** investigation, project administration. **Nurul Husna Mohd Dani:** investigation, project administration. **Zurina Zainuddin:** conceptualization, supervision.

## Funding

The authors have nothing to report.

## Disclosure

To protect the patient's identity, all personal identifiers have been removed. No identifiable information is included in this case report. Details that could potentially lead to the identification of the patient have been altered to ensure confidentiality. All data and images used in this case report are securely stored and accessible only to authorized personnel.

## Ethics Statement

All efforts have been made to ensure that the patient's privacy and confidentiality are maintained throughout the report. The information provided is strictly anonymized to prevent the identification of the patient.

## Consent

Informed and written consent was obtained from the patient's legal guardian for the publication of this case report and any accompanying images. The patient was informed about the purpose of the report, the information to be disclosed, and the potential implications of publication. The guardian has agreed to the use of their anonymized data for educational and research purposes.

## Conflicts of Interest

The authors declare no conflicts of interest.

## Data Availability

The data supporting the findings of this case report are contained within the article. No additional data are available due to patient confidentiality and privacy considerations.
